# Fire boundaries of lithium-ion cell eruption gases caused by thermal runaway

**DOI:** 10.1016/j.isci.2021.102401

**Published:** 2021-04-07

**Authors:** Weifeng Li, Shun Rao, Yang Xiao, Zhenhai Gao, Yupeng Chen, Hewu Wang, Minggao Ouyang

**Affiliations:** 1 State Key Laboratory of Automotive Simulation and Control, Jilin University, Changchun 130025, China; 2 Key Laboratory of Bio-inspired Smart Interfacial Science and Technology of Ministry of Education, School of Chemistry, Beihang University, Beijing 100191, P.R. China; 3 State Key Laboratory of Automotive Safety and Energy, Tsinghua University, Beijing 100084, PR China

**Keywords:** Electrochemistry, Electrochemical Energy Storage, Engineering, Mechanical Engineering

## Abstract

Lithium-ion batteries are applied in electric vehicles to mitigate climate change. However, their practical applications are impeded by poor safety performance owing mainly to the cell eruption gas (CEG) fire triangle. Here, we report quantitatively the three fire boundaries corresponding to the CEG fire triangle of four types of mainstream cells with the state of charge (SOC) values ranging from 0% to 143% based on 29 thermal runaway tests conducted in an inert atmosphere in open literature. Controlling the SOC and/or selecting a reasonable cell type can alter the minimum CEG and oxygen concentrations required for ignition, thereby changing the probability of a battery fire. The ignition temperature varies greatly according to the type of ignition source type. Temperature and ignition source type play a leading role in the ignition mode. Breaking any fire boundary will stop the ignition of CEG, thus significantly improving the battery safety performance.

## Highlights

• We clarified the three fire boundaries of LIBs corresponding to the fire triangle
• Batteries are prone to ignition with forced ignition sources
• Batteries are hard to autoignite when temperatures are low enough
• LIB ignition modes can be controlled by changing temperatures and ignition sources

## Graphical abstract

**Figure:**
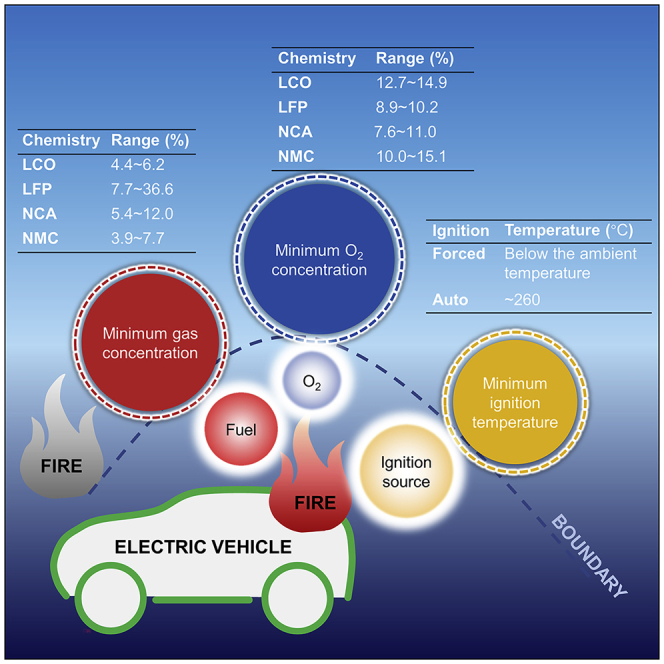

## Introduction

Electric vehicles are paid much attention to mitigate climate change (Stephan et al., 2021; Han et al., 2019; Gourley et al., 2020). After many years of development, lithium-ion batteries (LIBs) have become increasingly acceptable as the main power source of electric vehicles, given their higher energy density and longer life cycle (EIA, 2020; Liu et al., 2018). However, the safety aspects concerning electric vehicles have received increasing attention due to the hazards of possible fires, usually caused by the failure of on-board large capacity power batteries (Sun et al., 2020).

As one of the main energetic failures, thermal runaway refers to the rapid self-heating of a cell, resulting from the exothermic chemical reaction between the highly oxidizing positive electrode and highly reducing negative electrode of the cell. This can occur in batteries with almost any chemistry (Mikolajczak et al., 2011). With the occurrence of LIB thermal runaway, more and more gases are generated inside the cells. Then, when the pressure inside a cell reaches a certain value, the cell's safety valve is released, or the area at the aluminum-plastic film with the lower allowable pressure for the pouch cell develops a crack. Then, the cell erupts and releases gaseous emissions, i.e., cell eruption gases (CEGs) (Finegan et al., 2015; Wang et al., 2019a; Li et al., 2019b; Zhang et al., 2019). These gases are among the main combustion materials that lead to fires (Xu and Hui, 2017; Bi et al., 2015).

Because CEGs are generally released from the inside of a cell to the battery pack and the external environment, the main combustion-supporting material is oxygen (O_2_) in air. The parameters corresponding to the first two boundaries are the lower flammability limit (LFL) and upper flammability limit (UFL) of the CEGs, which are expressed by the CEG concentration in the CEG-air mixture. When the CEG concentration is lower than the LFL, the CEGs are too thin for ignition. Therefore, the LFL is the c_CEG, ignition_. When the CEG concentration is greater than the UFL, because it is too rich, meaning that the surrounding O_2_ is too thin, ignition cannot occur. The O_2_ concentration in the CEG-air mixture corresponding to the UFL is the minimum O_2_ concentration (c_O2, ignition_) required for ignition. When the CEG concentration is between the LFL and UFL, there is neither a lack of fuel nor O_2_ and ignition can occur.

It should be noted that the c_O2, ignition_ mentioned here refers to the O_2_ concentration in the CEG-air mixture at the LFL (Liu et al., 2004). It is due to the too rich fuel and too lean O_2_ for ignition to take place. Another similar concept is the critical O_2_ concentration (Fairweather et al., 1999), which refers to the O_2_ concentration in the fuel-air dilution mixture when the LFL coincides with the UFL using inert gas to dilute the fuel-air mixture. In fact, the critical O_2_ concentration is a special case of c_O2, ignition_.

To obtain the flammability limit of the CEG, three research methods are generally used. In the first method, thermal runaway is triggered in an inert atmosphere until eruption, and the CEG components are then detected. Afterward, calculations are performed on the basis of the detected components. Based on the existing results (Somandepalli et al., 2014), Guo et al. (Guo and Zhang., 2016) calculated the flammability limits of CEGs and found that the flammability range increases with an increase in the state of charge (SOC). In our open study (Li et al., 2019b), the flammability limits of the CEGs released by commercial 18,650 LIBs with lithium nickel cobalt aluminum oxide (NCA) and lithium iron phosphate (LFP) cathodes at 0%–143% SOCs were calculated using available data in open literature (Golubkov et al., 2015). We found that the UFL and LFL curves of CEGs form a peninsula shape for both cell types with a decrease in the SOC, where the flammability range did not essentially change at first and then dramatically decreased. For the LFP cell, the LFL of the CEGs was higher, and the flammability range was lower than that of the NCA cell at the same SOC.

In the second method, a thermal runaway test is performed in a vacuum environment, and the released cell gases are collected. Then, the flammability limit of the gases is directly tested through an experimental method using a combustion chamber. By using this method, Somandepalli et al. (Somandepalli et al., 2014) found that the LFL of CEGs is about 6.3% and the UFL is between 30 and 40% for cases of 100% and 150% SOCs.

The third method is similar to the second but is conducted in an air environment. In this case, the detected gas is the product of the reaction between CEGs and the air in the test container rather than the CEGs alone. However, the results are of important reference value for evaluating whether CEGs are flammable in the atmosphere after being released from battery packs. Long et al. (Long et al., 2014) subjected a 100 Ah 3.3 V cell to thermal runaway by overcharging it and then collected the CEGs. They opened the valve of the gas collection bag, ignited the gas using an igniter in a laboratory, and found that the CEGs continued to burn. Chen et al. (Chen et al., 2020) used a cell in a closed container filled with air to conduct a thermal runaway test and then tested the LFL of the CEG. They found that the LEL of the CEG increased at the initial stage and then decreased with an increase in the SOC. Moreover, they reported that batteries should be stored at 60% SOC in non-extremely dry environments to reduce the risk of explosion and that keeping the SOC at 100%, which has the lowest LEL, poses a high risk of danger caused by thermal runaway.

### However, some problems remain regarding cell eruptions and fires

First, there are few comparisons of the c_CEG, ignition_ for different types of cells, which makes it difficult to provide better guidance for cell selection and battery pack design. Baird et al. (Baird et al., 2020) evaluated the LFL of CEGs to quantify the cell chemistry effect and SOC using three modeling methods. They found that the LFL was 7.6–9.0, 8.6–10.0, 6.1–8.8, and 6.7–11.8 for lithium nickel cobalt manganese oxide (NMC), LFP, lithium cobalt oxide (LCO), and NCA cells, respectively. The results showed that the CEG of LFP generally had higher LFL values at 100% SOC, allowing for more gases to accumulate before reaching deflagration or a fire hazard compared with that of NCA or LCO cells. However, these calculation results were based on gases detected in air, vacuum, and inert atmospheres. It is difficult to distinguish which results were based on the CEG and which results were based on the reaction products of the CEG and air. CEGs are generally ejected from the inside of a cell to the battery pack and subsequently react with the air in the pack before being released to the atmosphere. Therefore, it is still difficult to directly provide guidance for the design of battery packs based on these results.

Second, insufficient data are available (Garche and Brandt, 2018) on the minimum O_2_/air concentration (without the introduction of other inert gases) required for CEG ignition for different types of cells. This makes it difficult to provide better guidance for battery pack design. If the amount of air inside a battery pack can be changed to make the O_2_ content below the c_O2, ignition_, ignition can be avoided, thus slowing the spread of heat and the resultant damage to the pack components, cells, circuits, and other parts.

Third, few analysis results (Garche and Brandt, 2018) have been presented for T _ignition_. If this boundary is known, the CEG temperature can be reduced to a value below the boundary through thermal management, thus avoiding the possibility of CEG ignition after their release.

Therefore, based on our previous research on the generation reasons (Li et al., 2019a), eruption characteristics (Wang et al., 2019a; Zhang et al., 2020), component identification (Zhang et al., 2019), ignition sources (Zhang et al., 2019), and flammability analyses (Li et al., 2019b) of CEGs, we summarize the CEG component identification results of 29 thermal runaway tests conducted in an inert atmosphere, as presented in the literature (Zhang et al., 2019; Somandepalli et al., 2014; Golubkov et al., 2014, 2015; Lammer et al., 2017; Essl et al., 2020). According to the results, a time sequence diagram of CEG generation is drawn, and the three fire boundaries of CEGs, including c_CEG, ignition_, c_O2, ignition_, and T _ignition_, are analyzed on the basis of thermal ignition theory. Overall, this research can provide theoretical guidance for cell selection, pack design, and fire safety design.

### Review of the cell eruption gas components

This study focuses on summarizing the performed works (Zhang et al., 2019; Somandepalli et al., 2014; Golubkov et al., 2014, 2015; Lammer et al., 2017; Essl et al., 2020) in the last 10 years regarding the identification of CEG in an inert atmosphere because triggered thermal runaway in an inert atmosphere avoids chemical changes as much as possible after the CEG is ejected from the cell.

Table 1 shows equipment used in the summarized works (Zhang et al., 2019; Somandepalli et al., 2014; Golubkov et al., 2014, 2015; Lammer et al., 2017; Essl et al., 2020) and the types of gases detected. The used instruments mainly included gas chromatography-mass spectrometers (GC-MSs), gas chromatographers (GCs), thermal conductivity detectors (TCGs), ion chromatographs (ICs), and Fourier transform infrared spectrometers (FTIRs). The types of detected gases mainly included hydrogen (H_2_), oxygen (O_2_), nitrogen (N_2_), carbon monoxide (CO), carbon dioxide (CO_2_), methane (CH_4_), ethyne (C_2_H_2_), ethylene (C_2_H_4_), ethane (C_2_H_6_), and other hydrocarbons. In addition, diethyl carbonate (DEC), methyl ethyl carbonate (EMC), dimethyl carbonate (DMC), hydrogen chloride (HCl), ethylene carbonate (EC), hydrogen fluoride (HF), etc., were also detected.

**Table 1:** Equipment used to detect the cell eruption gases in the summarized works

Literature	Equipment	Model	Gas detected
Somandepalli et al. (2014)	GC-MS	–	CO, CO_2_, H_2_, and hydrocarbons
Golubkov et al. (2014)	GC	Agilent 3000 Micro GC, two columns, Mol Sieve and PLOTU	H_2_, O_2_, N_2_, CO, CO_2_, CH_4_, C_2_H_2_, C_2_H_4_, and C_2_H_6_
	TCD	–	Permanent gases
Golubkov, et al., 2015	GC	Agilent 3000 Micro GC, two columns, Mol Sieve and PLOTU	H_2_, O_2_, N_2_, CO, CO_2_, CH_4_, C_2_H_2_, C_2_H_4_, and C_2_H_6_
	TCG	–	Permanent gases
Lammer et al., 2017	GC	Agilent Micro-GC 3000A	H_2_, CO, CO_2_, CH_4_, C_2_H_2_, C_2_H_4_, and C_2_H_6_
Zhang et al., 2019	GC	Agilent 7890A	H_2_, CO, CO_2_, and hydrocarbons
	GC-MS	Agilent 7890B-5977A	DEC, EMC
	IC	Metrohm 930 Compact	HCl
Essl et al., 2020	FTIR	Bruker MATRIX-MG01	CO, CO_2_, CH_4_, C_2_H_6_, C_2_H_4_, C_2_H_2_, DEC, DMC, EC, EMC, H_2_O, C_6_H_14_, HF, C_4_H_10_, and C_3_H_8_
	GC	3000 Micro GC (G2802A) with three columns and TCD detectors	H_2_, O_2_, N_2_, CH_4_, CO, CO_2_, C_2_H_6_, C_2_H_4_, C_2_H_2_

For more information, refer to Zhang et al. (2019); Somandepalli et al. (2014); Golubkov et al. (2015); Golubkov et al. (2014); Lammer et al. (2017); and Essl et al. (2020).

Table 2 shows the details of the cells used in the summarized works (Zhang et al., 2019; Somandepalli et al., 2014; Golubkov et al., 2014, 2015; Lammer et al., 2017; Essl et al., 2020). The cell chemistries include common types, such as LCO, LPF, NCA, and NMC. The cell capacity ranged from 1.1 Ah to 50 Ah, and the cell formats included square, 18650, and pouch. The SOC values varied from 0% to 143%.

**Table 2:** Details of the cells used in the thermal runaway tests in inert atmosphere in the summarized works

Test no.	Literature	Legend	Chemistry	Format	Nominal capacity (Ah)	SOC (%)
1	Somandepalli et al. (2014)	LCO_2.1 Ah	LiCoO_2_	–	2.1	50
2						100
3						150
4	Golubkov et al. (2014)	LFP_1.1 Ah (2014)	LiFePO_4_	18650	1.1	100
5	Golubkov, et al., 2015	LFP_1.1 Ah (2015)	Li_0.882_FePO_4_	18650	1.1	0
6						25
7						50
8						75
9						100
10						115
11						130
12	Lammer et al. (2017)	NCA_3.2 Ah	LiNi_0.8_Co_0.15_Al_0.05_O_2_	18650	3.2	100
13	Golubkov et al. (2015)	NCA_3.35 Ah	Li_0.925_(Ni_0.80_Co_0.15_Al_0.05_)O_2_	18650	3.35	0
14						25
15						50
16						75
17						100
18						112
19						120
20						127
21						132
22						143
23	Lammer et al. (2017)	NCA_3.5 Ah (47.68 g)	LiNi_0.8_Co_0.15_A_l0.05_O_2_	18650	3.5	100
24		NCA_3.5 Ah (46.35 g)				100
25	Golubkov et al. (2014)	NMC_1.5 Ah	Li(Ni_0.45_Mn_0.45_Co_0.10_)O_2_	18650	1.5	100
26	Zhang et al. (2019)	NMC_50 Ah	Li(Ni_0.6_Mn_0.2_Co_0.2_)O_2_	Prismatic	50	100
27	Golubkov et al. (2014)	NMC/LCO_2.6 Ah	LiCoO_2_/Li(Ni_0.50_Mn_0.25_Co_0.25_)O_2_	18650	2.6	100
28	Essl et al. (2020)	NMC/LMO_41Ah	LiNiMnCoO_2_/LiMn_2_O_4_	Pouch	41	100
29						30

For more information, refer to Zhang et al. (2019); Somandepalli et al. (2014); Golubkov et al. (2015); Golubkov et al. (2014); Lammer et al. (2017); and Essl et al. (2020).

**Figure 1:**
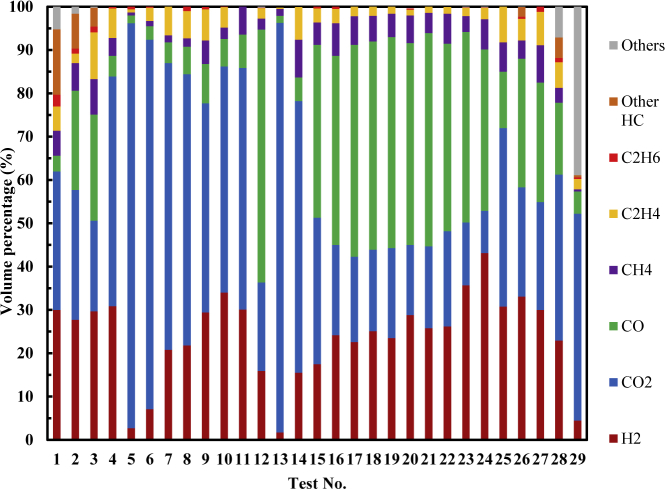
Variations of the volume percentage of the CEG components in the summarized works vs. test number CEG identification result is based on 29 thermal runaway tests conducted in an inert atmosphere in open literature.

Figure 1 shows the main CEG components detected in the summarized works (Zhang et al., 2019; Somandepalli et al., 2014; Golubkov et al., 2014, 2015; Lammer et al., 2017; Essl et al., 2020), which were H_2_, CO_2_, CO, CH_4_, C_2_H_4_, and C_2_H_6_. In addition, the components included electrolyte vapor, HF, and other gases. The formation reactions of the main CEG components are summarized in detail in the study by (Wang et al., 2019b).

**Figure 2:**
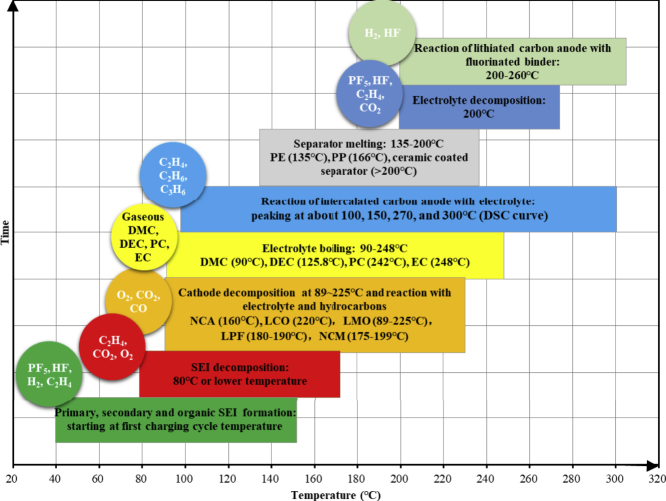
Time sequence of CEG generation Temperature without special explanations refers to the onset temperature of reaction, decomposing, boiling, or melting.

Figure 2 shows the time sequence of the CEG generation. In addition to the electrolyte vaporization (90°C–248°C) caused by physical changes, the CEG also contains new gases generated by chemical reactions, which can be explained by the thermal decomposition and reactions of the electrolyte, binder, and electrode materials (Golubkov et al., 2014; Wang et al., 2012; Roth and Orendorff., 2012; Fleischhammer and Döring., 2013; Pfrang et al., 2017), as mentioned in the summarized works (Golubkov et al., 2014, 2015; Kocha et al., 2018).

The solid electrolyte interphase (SEI) is a reaction layer that is formed by electrolyte reduction during the first charging cycle on the surfaces of carbon-based anodes (Garche and Brandt, 2018). During the formation of the primary SEI, gases including phosphorus pentafluoride (PF_5_), HF, H_2_, C_2_H_4_, etc., are produced (Agubra and Fergus, 2014; Aurbach et al., 1999; Watanabe and Yamaki, 2006). In general, the SEI consists of inorganic and organic compounds. The organic compounds are metastable at around 80°C, and they start to react and form the so-called secondary SEIs (Wang et al., 2006; Yang et al., 2005; Richard and Dahn, 1999; Andersson and Edström, 2001). The secondary SEI mainly consists of lithium carbonate (Li_2_CO_3_) and lithium fluoride (LiF) (Yang et al., 2005). It has been suggested that besides the formation of secondary SEIs, new organic SEIs are formed by solvent reduction. These complex processes of SEI formation and change occur up to a temperature of ∼200°C (Wang et al., 2006; Zhou et al., 2012). During the formation of secondary SEIs, gases including HF, C_2_H_4_, CO_2_, O_2_, C_2_H_4_, etc., are produced (Agubra and Fergus, 2014; Aurbach et al., 1999; Zhou et al., 2012). The initial decomposition of SEI occurs at 80°C–120°C (Spotnitz and Franklin, 2003) with a peak at ∼100°C (Richard and Dahn, 1999). An SEI layer may decompose at relatively lower temperatures, such as 69°C (Wang et al., 2006) or 57°C (Wang et al., 2005). C_2_H_4_, CO_2_, O_2_, and other gases are produced during the thermal decomposition of SEI (Yang et al., 2005).

The differential scanning calorimetry traces of the lithiated carbon anodes and electrolytes become very complex at the following peaks: ∼100°C, ∼150°C, ∼270°C, and ∼300°C (Spotnitz and Franklin, 2003).

Organic solvents (EC, PC, DMC, etc.) can also react with intercalated lithium to release flammable hydrocarbons, such as C_2_H_4_, C_3_H_6_, and C_2_H_6_ (Spotnitz and Franklin, 2003; Aurbach et al., 1997; Gachot et al., 2010, 2012; Yoshida et al., 1997; Onuki et al., 2008; Shin et al., 2002).

The PE and PP separators melt at 135°C and 166°C, respectively, while some ceramic-coated separators may maintain their structural integrity even above 200°C (Mao et al., 2018; Orendorff, 2012). It has not been previously reported in open literature that gas can be produced during this process.

The initial decomposition of cathodes occurs at 89°C–225°C (Biensan et al., 1999; Wang et al., 2007a, 2007b; Huang et al., 2016; Zhang et al., 1998; Martha et al., 2011; Joachin et al., 2009), and then, O_2_ is released (Dahn et al., 1994; Li et al., 2006). The release of O_2_ can lead to a further reduction of the generated hydrocarbons down to CO_2_. Since this O_2_ generation from the cathodes inside the cells and the other O_2_ sources are both limited, some hydrocarbons only get reduced to CO (Golubkov et al., 2014; Roth and Orendorff., 2012).

LiPF_6_ salt decomposes at 200°C to LiF and PF_5_ (Ravdel et al., 2003). The decomposition of the electrolyte is a multistage reaction and mainly takes place in the ranges of 200°C–220°C, 220°C–250°C, and 250°C–300°C, generating gases such as PF_5_, HF, CO_2_, and C_2_H_4_ (Ribiere et al., 2012; Wang et al., 2019b; Campion et al., 2004; Gnanaraj et al., 2003; Kawamura et al., 2006).

When a carbon anode is intercalated with lithium-ions, it can react with PVDF, generating HF and H_2_ (Pasquier et al., 1998). The temperatures at which the reaction begins were reported to be 200°C (Maleki et al., 1999), 240°C (Biensan et al., 1999), and 260°C (Pasquier et al., 1998).

## Results and discussion

Gas can be divided into two types: non-flammable and flammable. In the former case, no gas ignition will occur regardless of the conditions. As determined in tests 5 and 13 shown in Table 2 and Figure 1, CEGs are non-flammable when the SOC is 0% owing to the high CO_2_ content (Li et al., 2019b). However, the CEGs were flammable in the other 27 tests. It should be noted that flammable does not guarantee ignition. To achieve fire, combustibles need an oxidizer, an ignition source, ignition energy, ignition critical diameter, etc (Xu and Hui, 2017; Bi et al., 2015; Turns and Haworth, 2021). The main conditions for ignition are collectively known as the fire triangle, i.e., a combustible, an oxidizer, and an ignition source. The three fire boundaries corresponding to the fire triangle are c_CEG, ignition_, c_O2, ignition_ and T _ignition_. According to the thermal ignition theory, these three boundaries are necessary for fire but not sufficient (Xu and Hui, 2017; Bi et al., 2015; Turns and Haworth, 2021). When one of the fire boundaries is met, a fire may occur or not. But when any one of the fire boundaries is not met, a fire cannot occur. This means that if any one of fire boundaries is broken, no fire will occur. This is of great significance for battery fire suppression. This section analyzes the three fire boundaries of flammable CEGs in a cell fire based on the thermal ignition theory. When analyzing the impact of a certain boundary, it is assumed that the other fire boundaries are available. Considering the limited amount of data in open literature (Zhang et al., 2019; Somandepalli et al., 2014; Golubkov et al., 2014, 2015; Lammer et al., 2017; Essl et al., 2020), when discussing the changes in c_CEG, ignition_, and c_O2, ignition_ with SOC, only the trends of LFP_1.1 Ah (2015), NCA_3.35 Ah (2015), and LCO_2.1 Ah were discussed. In addition, to compare the differences between cell types, cells using NMC, NMC/LCO, and NMC/LMO as positive electrodes were collectively classified as NMC cells.

### Minimum CEG concentration required for ignition

**Figure 3:**
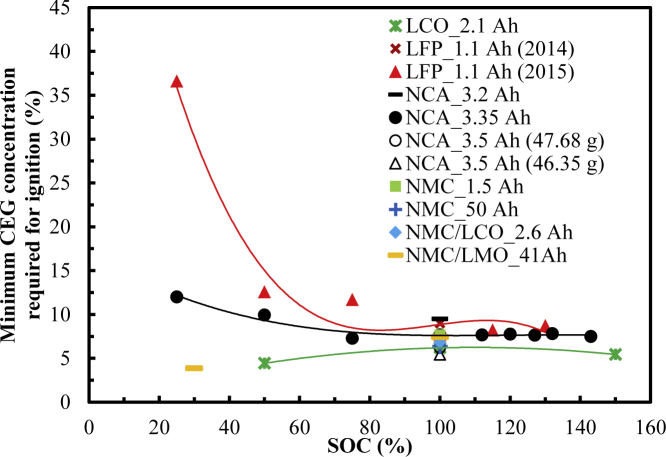
Variations in the minimum CEG concentration required for ignition vs. SOC

Figure 3 shows the variation in c_CEG, ignition_ with the SOC for different types of cells. The calculation method of c_CEG, ignition_ is shown in the supplemental information section. It decreases with an increase in the SOC for the LFP_1.1 Ah (2015) cell at the discharged state, especially when the SOC is below 50%. This shows that the probability of fire increases with the SOC value. Also, c_CEG, ignition_ remains almost unchanged at the full and overcharged stages. However, from the discharged (25% SOC) to the fully charged (100% SOC) to the overcharged (130% SOC) stages, it successively decreases by 79.0% and increases by 13.0%.

Compared with c_CEG, ignition_ for the LFP_1.1 Ah (2015) cell, c_CEG, ignition_ for the NCA_3.35 Ah (2015) cell has a similar variety trend with an increase in the SOC. From the discharged (25% SOC) to the fully charged (100% SOC) to the overcharged (143% SOC) stages, it successively decreases by 35.8% and increases by 2.6%.

For the LCO_2.1 Ah cell, c_CEG, ignition_ first increases and then slightly decreases with an increase in the SOC. From the discharged (25% SOC) to the fully charged (100% SOC) to the overcharged (143% SOC) stages, it successively increases by 40.9% and decreases by 12.9%.

Thus, for these three types of cells, at the same SOC, the LFP_1.1 Ah (2015) cell requires the highest c_CEG, ignition_, followed by the NCA_3.35 Ah (2015) cell and then the LCO_2.1 Ah cell. It successively decreases by 21.4% and 55.6% at the discharged state (50% SOC). Then, it successively decreases by 0.8% and 19.5% at the fully charged state (100% SOC). This shows that when the other fire conditions are the same, the LFP_1.1 Ah (2015) cell has the lowest fire possibility, followed by the NCA_3.35 Ah (2015) cell and then the LCO_2.1 Ah cell.

**Figure 4:**
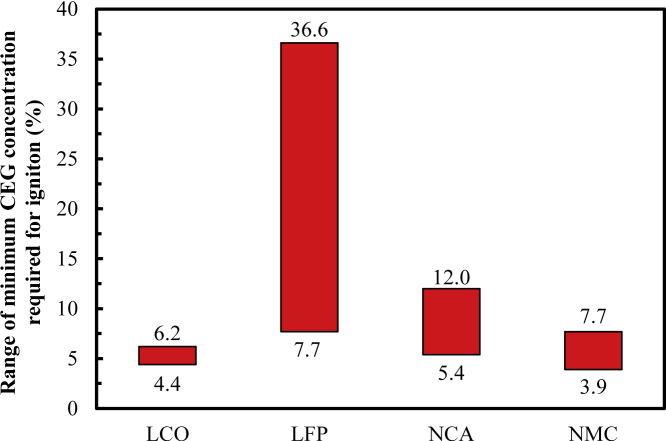
Variations in the range of the minimum CEG concentration required for ignition vs. cell type

Table 3 shows the range of c_CEG, ignition_ under different charging states. The respective c_CEG, ignition_ for the LCO, LFP, NCA, and NMC cells is 4.4%, 11.7%–36.3%, 7.3%–12.0%, and 3.9% when not fully charged and 6.2%, 7.7%, 5.4%–9.5%, and 6.4%–7.7% for the case of being fully charged, respectively. When LCO, LFP and NCA are overcharged, the values are 5.4%, 8.2%–8.7%, and 7.5%–7.9%, respectively. Overall, the c_CEG, ignition_ for the LCO, LFP, NCA, and NMC cells is 4.4%–6.2%, 7.7%–36.6%, 5.4%–12.0%, and 3.9%–3.9%, respectively. The c_CEG, ignition_ for the LFP cell is highest, followed by the NCA and LCO cells and then the NMC cell, as shown in Figure 4. This shows that the fire probability for these types of cells successively increases and that the difficulty of their fire suppression by controlling the CEG concentration also successively increases.

**Table 3:** Minimum CEG concentration required for ignition for different cell types.

Chemistry	Not fully charged	Fully charged	Overcharged	Range
LCO	4.4	6.2	5.4	4.4–6.2
LFP	11.7–36.6	7.7	8.2–8.7	7.7–36.6
NA	7.3–12.0	5.4–9.5	7.5–7.9	5.4–12.0
NMC	3.9	6.4–7.7	–	3.9–7.7

The above analysis results show that by controlling the SOC and/or selecting a reasonable cell type, the c_CEG, ignition_ of a cell can be changed, thereby changing the probability of battery fire.

### Minimum O_2_ concentration required for ignition

**Figure 5:**
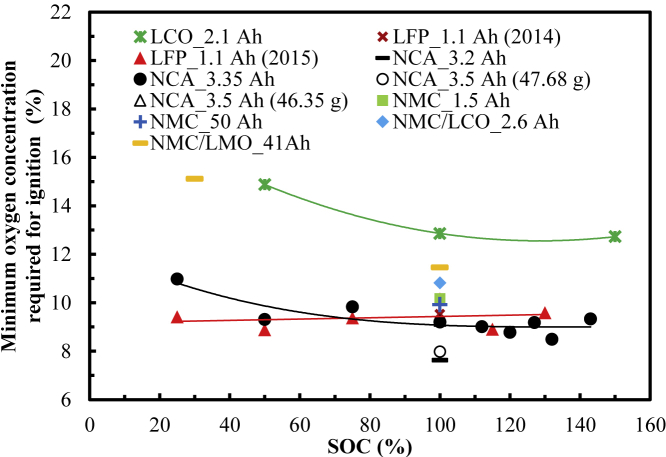
Variations in the minimum oxygen concentration required for ignition vs. SOC

Figure 5 shows the variation in c_O2, ignition_ with the SOC for different types of cells. The calculation method of c_O2, ignition_ is shown in the supplemental information section. For the LFP_1.1 Ah (2015) cell, as the SOC value increases, it does not significantly change. From the discharged (25% SOC) to the fully charged (100% SOC) to the overcharged (130% SOC) stages, it successively increases by 8.5% and decreases by 5.9%.

For the NCA_3.35 Ah (2015) cell, as the SOC value increases, c_O2, ignition_ decreases at the discharged state but remains almost unchanged at the fully charged and overcharged stages. From the discharged (25% SOC) to the fully charged (100% SOC) to the overcharged (143% SOC) stages, it successively decreases by 16.4% and increases by 1.1%.

The LCO_2.1 Ah cell has a similar trend to that of the NCA_3.35 Ah (2015) cell. From the discharged (50% SOC) to the fully charged (100% SOC) to the overcharged (150% SOC) stages, c_O2, ignition_ successively decreases by 13.4% and increases by 1.6%.

For these three types of cells, at the same SOC, the LCO_2.1 Ah cell requires higher c_O2, ignition_ than that of the other two cell types. For the same SOC value, the NCA_3.35 Ah (2015) cell requires higher c_O2, ignition_ than that of the LFP_1.1 Ah (2015) cell at the discharged state. However, there is no obvious difference in c_O2, ignition_ at the fully and overcharged states for these two cells. From the LCO_2.1 Ah cell to the NCA_3.35 Ah (2015) cell to the LFP_1.1 Ah (2015) cell, c_O2, ignition_ successively decreases by 37.6% and 4.3% at the discharged state (50% SOC) and successively decreases by 30.2% and increases by 10.9% at the fully charged state (100% SOC), respectively. This shows that when the other fire conditions are the same, the LCO_2.1 Ah cell has the lowest fire possibility among these three types of cells.

**Figure 6:**
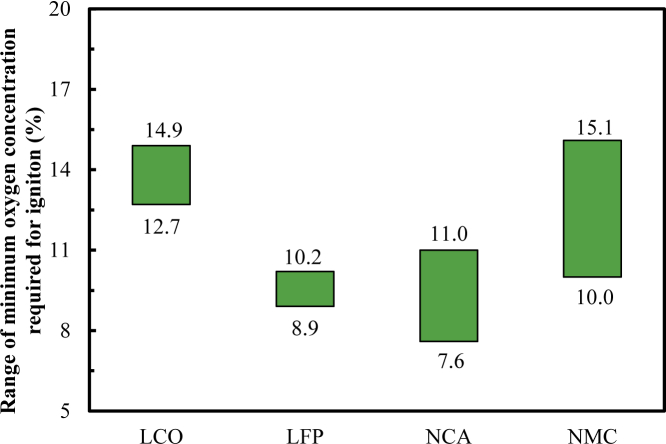
Variations of the range of the minimum O_2_ concentration required for ignition vs. cell type

Table 4 shows the range of c_O2, ignition_ under different charging states. For the LCO, LFP, NCA, and NMC cells, the respective values are 14.9%, 8.9%–9.4%, 9.3%–11.0%, and 15.1% for the case of being not fully charged and 12.9%, 10.2%, 7.6%–9.3%, and 10.0%–11.5% when fully charged, respectively. For the overcharged LCO, LFP, NCA cells, the values are 12.7%, 8.9%–9.6%, and 8.5%–9.3%, respectively. In general, c_O2, ignition_ for the LCO, LFP, NCA, and NMC cells is 12.7%–14.9%, 8.9%–10.2%, 7.6%–11.0%, and 10.0%–15.1%, respectively. Thus, the LCO cell requires the highest c_O2, ignition_ to ignite, followed by the NMC and LFP cells and then NCA cell, as shown in Figure 6. This shows that the fire hazard of these types of cells increases in turn and that the difficulty of their fire suppression by controlling the O_2_ concentration also successively increases.

**Table 4:** Minimum oxygen concentration required for ignition for different types of cells.

Chemistry	Not fully charged	Fully charged	Overcharged	Range
LCO	14.9	12.9	12.7	12.7–14.9
LFP	8.9–9.4	10.2	8.9–9.6	8.9–10.2
NA	9.3–11.0	7.6–9.3	8.5–9.3	7.6–11.0
NMC	15.1	10.0–11.5	–	10.0–15.1

The above analysis results show that by controlling the SOC and/or selecting a reasonable cell type, the c_O2, ignition_ of the cell can be changed, thereby changing the probability of battery fire.

It should be noted that the results of evaluating the cell safety based on c_CEG, ignition_ and c_O2, ignition_ are different. Based on the former, the order of safety from high to low is LFP > NCA > LCO > NMC. Based on the latter, the order of safety from high to low is LCO > NMC > LFP > NCA. This shows that a cell should be selected based on its application; for different types of cells, different fire prevention and control strategies should be selected.

The higher the c_CEG, ignition_ , the easier it is to suppress battery fire by controlling the CEG concentration. The same case applies for c_O2, ignition_. For example, c_CEG, ignition_ for NMC cells is relatively low, while c_O2, ignition_ is relatively high. This shows that to suppress NMC battery ignition, it is easier to control the O_2_ concentration than to control the CEG concentration. From the perspectives of c_CEG, ignition_ and c_O2, ignition_ for four different types of cells, to achieve fire suppression, it is recommended to control the CEG concentration for the LFP and NCA cells and the O_2_ concentration for the LCO and NMC cells.

However, actual scenarios should also be considered to select appropriate control methods. For example, for the inside of a closed battery box, the CEG and O_2_ concentrations can be reduced by filling incombustible gas or the O_2_ concentration can be reduced by reducing the internal pack space (after a cell erupts). It is difficult to control the O_2_ concentration in the atmosphere, so it should be mixed with incombustible gas before CEGs are released and reduced to a value below c_CEG, ignition_ to avoid fires.

Notably, because c_O2, ignition_ refers to the concentration of O_2_ in the CEG-air mixture, it is lower than the O_2_ content in the air (21%). In an open environment, sufficient air will continuously dilute the flammable CEG and can easily meet the O_2_ concentration boundary (Xu and Hui, 2017; Bi et al., 2015; Turns and Haworth, 2021). Therefore, if all other fire conditions are met, a fire will occur in an open environment. However, this does not mean that all CEGs will ignite in air because some CEGs are nonflammable (test 5 and 13 shown in Table 2 and Figure 1). In a closed environment, such as inside a battery box or a closed battery transport space, it is easier to control the O_2_ content. The O_2_ concentration boundary can be broken by reducing the amount of air by lowering the pressure, reducing the volume, and filling with inert gas to avoid the occurrence of fire (Li et al., 2019b; Turns and Haworth, 2021; Chen et al., 2017; Xie et al., 2020; Dong et al., 2019).

### Minimum ignition temperature required for ignition

Table 5 shows the main components of CEGs in open literature (Zhang et al., 2019; Somandepalli et al., 2014; Golubkov et al., 2014, 2014, 2015; Golubkov et al., 2014; Essl et al., 2020). In addition to CO_2_, H_2_O, and O_2_, 33 flammable substances have been found so far, such as CO, H_2_, alkane, alkene, alkyne, aromatic HC, electrolyte, etc. Based on the substances marked with ∗, the ignition mode and T _ignition_ of cells were analyzed in this section.

**Table 5:** Main components of CEGs found in open literature

Category	No.	Name	Formular	Essl et al. (2020)	Zhang et al. (2019)	Lammer et al. (2017)	Golubkov et al. (2015)	Golubkov et al. (2014)	Somandepalli et al. (2014)
Non-HC	1	Carbon dioxide	CO_2_	√	√	√	√	√	√
	2	Carbon monoxide	CO	√	√∗	√	√	√	√
	3	Hydrogen	H_2_	√	√∗	√	√	√	√
Alkane	4	Methane	CH_4_	√	√∗	√	√	√	√
	5	Ethane	C_2_H_6_	√	√∗	√	√	√	√
	6	Propane	C_3_H_8_	√	√∗				√
	7	n-Butane	C_4_H_10_	√	√∗				√
	8	Isobutane	C_4_H_10_						√
	9	n-Pentane	C_5_H_12_		√∗				√
	10	Isopentane	C_5_H_12_						√
Alkene	11	Ethylene	C_2_H_4_	√	√∗	√	√	√	√
	12	Propylene	C_3_H_6_		√∗				
	13	1-Butylene	C_4_H_8_		√∗				√#
	14	2-Methyl propene	C_4_H_8_		√				√#
	15	trans-2-Butene	C_4_H_8_		√				√#
	16	cis-2-Butene	C_4_H_8_		√				√#
	17	1-Pentene	C_5_H_10_		√∗				
	18	cis-2-Pentene	C_5_H_10_		√				
	19	trans-2-Pentene	C_5_H_10_		√				
	20	2-Methyl-1-butene	C_5_H_10_		√				
	21	2-Methyl-2-butene	C_5_H_10_		√				
	22	3-Methyl-1-butene	C_5_H_10_		√				
	23	2-Methyl-1-pentene	C_6_H_12_		√∗				
Alkyne	24	Ethyne	C_2_H_2_	√	√∗	√			
	25	Propyne	C_3_H_4_		√∗				√
	26	1,3-Butadiene	C_4_H_6_		√∗				
Aromatic HC	27	Benzene	C_6_H_6_		√∗				√
	28	Methylbenzene	C_7_H_8_						√∗
	29	Ethylbenzene	C_8_H_10_						√∗
	30	m & p-xylene	C_8_H_10_						√
Electrolyte	31	DMC	C_3_H_6_O_3_		√∗				
	32	EMC	C_4_H_8_O_3_		√∗				
	33	DEC	C_5_H_10_O_3_	√	√∗				
Others	34	2,4-Dimethyl-1-heptene	C_9_H_18_		√∗				
	35	Oxidane	H₂O	√	√				
	36	Hydrogen chloride	HCl		√				
	37	Oxygen	O_2_	√					

∗ Substance was used to analyze the temperature boundary and ignition mode.

# The type of isomer cannot be determined.

For more information, refer to Essl et al. (2020); Zhang et al. (2019); Golubkov et al. (2014, 2015); and Somandepalli et al. (2014).

According to thermal ignition theory, the ignition of CEG is divided into forced ignition and autoignition, as shown in Table 6. Forced ignition signifies that the CEG is heated locally by forced ignition sources, and the local CEG ignites first. Then, the produced flame spreads from the ignition zone to the others. A forced ignition source often has high temperature. Common forced ignition sources include sparks, hot spots, and flames, as shown in Table 6. The electrification of automobiles creates conditions for the generation of electric sparks, and the maximum temperature of electric sparks can be close to 10,000°C. The minimum temperature required for a substance to be forced to ignite is defined as the forced ignition point (T _forced-ignition_).

**Table 6:** Ignition source and its temperature

	Definition	Ignition source		T_Ignition source_
				°C
Forced ignition	The CEG is heated locally by forced ignitions, and the local CEG ignites first, and then, the flame spreads to the others. Forced ignition sources often have high temperatures.	Spark	(1) Electric spark caused by too small electric clearance between conductive parts	3000–6000
			(2) Electric arc caused by lots of sparks	8700–9700
			(3) Static electric spark caused by invalid equipotential bonding	–
			(4) Mechanical spark caused by friction between the eruption flow and the wall	~1200
			(5) Spark from the ICE pipe	600–800
		Hot spot	6) High temperature surface of the cell	~1000
			(7) High temperature cable with short circuit or overcurrent	–
			(8) Cigarette butts	250–800
		Flame	(9) Gas flame	1600–2100
			(10) Gasoline flame	~1200
			(11) Match flame	500–650
Autoignition	The CEG is heated whole by autoignition sources and then ignites. The autoignition source does not need to have a high temperature but needs to have enough energy to heat the CEG.	Self-heating	(1) Heats from the chemical reactions during the generating process of CEGs	200–1000
			(2) Heats from slow chemical reactions of CEGs caused by lighting, catalytic reactions by cathode materials, etc.	–
		Non-self-heating	(3) Heats from high temperature autoignition sources often with indirect contact with the CEG, such as the high temperature surface of a cell with thermal runaway, the high temperature surface of the ICE of another vehicle, a heater, etc. They can make the temperature of all the CEG be increased.	–
			(4) An energy source that converts other forms of energy into heat, such as friction, compression, etc.	–

Autoignition signifies that all CEGs are heated by autoignition sources and then ignite. An autoignition source does not require a high temperature but needs to have enough energy to heat the CEG. According to the energy source, autoignition sources are divided into self-heating and nonself-heating sources, as shown in Table 6. The main difference between a nonself-heating source and a forced ignition source is whether the ignition source is in direct contact with combustibles, and whether it can increase the temperature of the overall combustibles. The lowest temperature required for a substance to spontaneously ignite without forced ignition sources is defined as the autoignition point (T _autoignition_).

Forced ignition and autoignition are essentially the same. After heat accumulates to a certain extent, the chemical reaction rate is automatically and continuously accelerated until a higher chemical reaction rate is reached. The main difference is that the former is local heating, and the latter is overall heating. To facilitate the analysis, the following assumptions were made:

a) T _forced-ignition_ is usually 5°C–20°C higher than the flash point (T _flash_, the minimum temperature required for a substance to flash), but the T _forced-ignition_ data are incomplete and are related mainly to testing methods and boundaries. Therefore, T _flash_ is used to measure the T _forced-ignition_ of CEG components.
b) The influences of the pressure and temperature inside a cell on the physical and chemical properties of the CEG components were not considered.
c) The cell jet area temperature was used to represent the CEG temperature during eruption.
d) For the convenience of analysis, it was considered that the CEG temperature, i.e., T _eruption_, is about 350°C (Zhang et al., 2019) and that the ambient temperature (T _ambient_) is ∼25°C.

**Figure 7:**
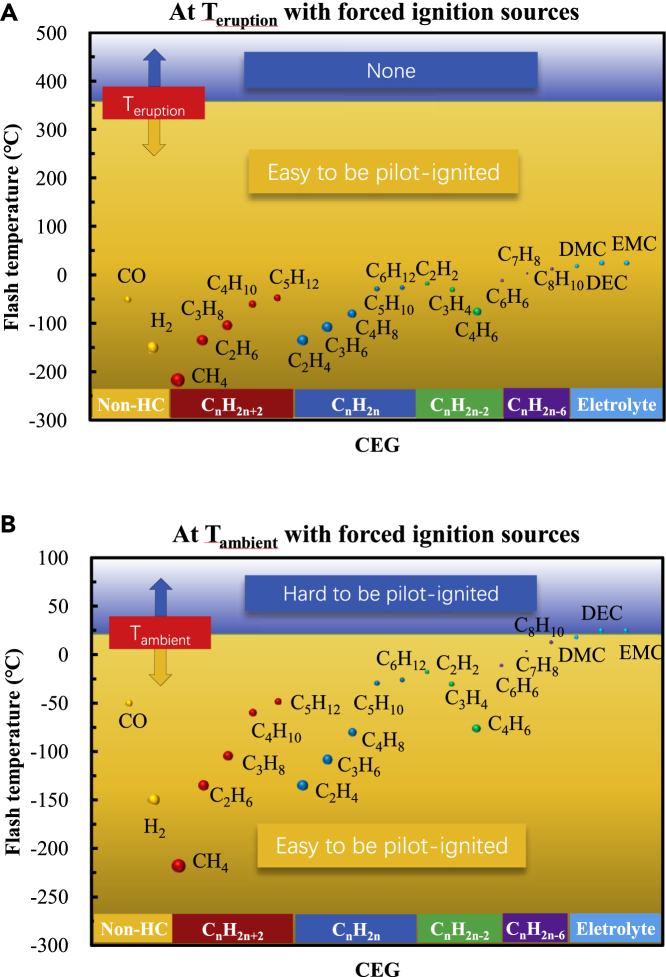
Flash temperatures of the main CEG components When there is a forced ignition source, the temperature boundary is T _flash_. That is, when the CEG temperature exceeds T _flash_, the CEG may be forced ignited. (A) When a cell erupts, the CEG is easily ignited if other ignition boundaries are available. (B) If the CEG is cooled to T _ambient_, substances with T _flash_ lower than T _ambient_ can easily ignite.

When there is a forced ignition source, the temperature boundary is T _flash_. That is, when the CEG temperature exceeds T _flash_, the CEG may be forced ignited. Figure 7 shows the T _flash_ of the CEG main components. As the number of carbon atoms increases, T _flash_ increases for alkanes (carbon atoms fewer than 6), alkenes (carbon atoms less than 7), and aromatic hydrocarbons (carbon atoms fewer than 9), but it decreases for alkynes (carbon atoms fewer than 5). The T _flash_ values of the three electrolytes are not significantly different. Among the detected substances, the substance with the lowest T _flash_ is CH_4_, which is around −200°C, and the substance with the highest T _flash_ is the electrolyte, which is higher than 0°C. When there is a forced ignition source, there are two typical situations:

a) When a cell erupts, the CEG is easily ignited if other ignition boundaries are available, as shown in Figure 7A, because the T _flash_ values of all of the substances are lower than T _eruption_ (about 350°C (Zhang et al., 2019)).
b) If the CEG is cooled to T _ambient_, substances with T _flash_ lower than T _ambient_ can easily ignite. Among the CEG components, CO, hydrogen, small molecular alkanes, small molecular olefins, and other substances generally have a flash point lower than the T _ambient_ (about 25°C), so they are easily ignited first. The electrolyte, macromolecular alkanes, macromolecular alkenes, small molecular alkynes, benzene, and other substances may have a higher flash point than T _ambient_ (e.g., cold winter), so these substances may be ignited by the other substances that were already ignited first, as shown in Figure 7B.

**Figure 8:**
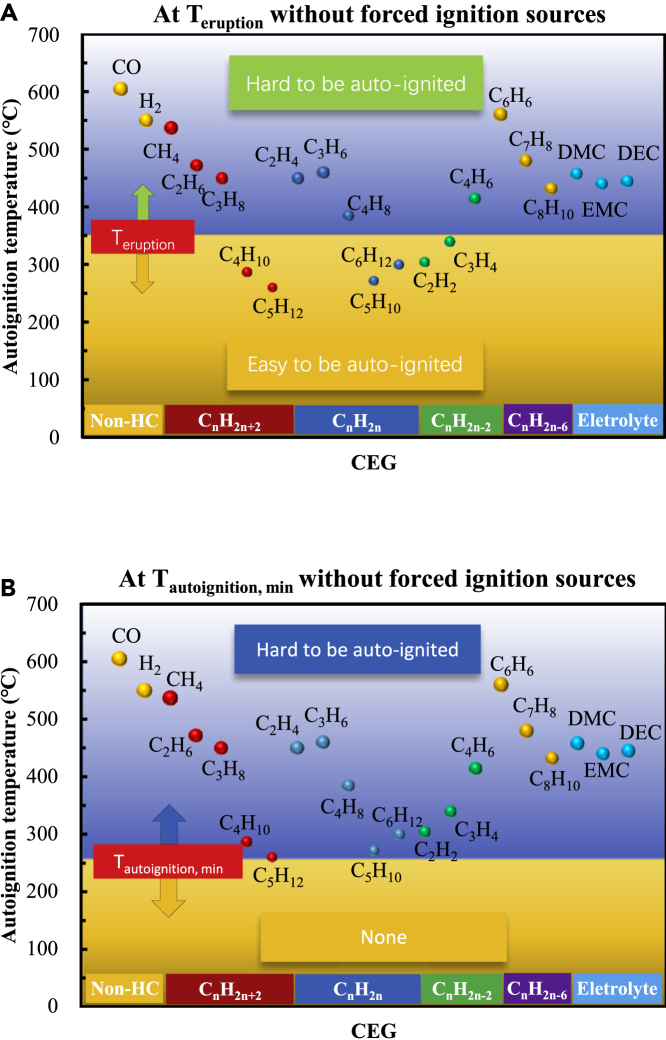
Autoignition temperatures of the main CEG components When there is no forced ignition source, the temperature boundary is T _autoignition_. That is, when the fuel temperature exceeds T _auto-ignition_, CEG may be autoignited. (A) When a cell erupts, the substances with T _auto-ignition_ lower than T _eruption_ are easy to autoignite first, and then, they ignite the substances with T _auto-ignition_ higher than T _eruption_. (B) If the CEGs are cooled below T _autoignition, min_ of ~260°C, autoignition will not occur.

When there is no forced ignition source, the temperature boundary is T _autoignition_. That is, when the fuel temperature exceeds T _auto-ignition_, CEGs may be autoignited. Figure 8 shows the T _autoignition_ of the main CEG components. For the alkanes (carbon atoms less than 6), alkenes (carbon atoms less than 7), and aromatic hydrocarbons (carbon atoms less than 9), as the number of carbon atoms increases, the overall T _autoignition_ shows a downward trend, but it increases for the alkynes (the number of carbon atoms is less than 5). Among the detected substances, CO has the highest T _autoignition_, followed by C_6_H_6_, H_2_ and CH_4_ (all above 500°C); the substances with lower T _autoignition_ (around 300°C) are mainly macromolecule alkanes (e.g., C_4_H_10_, C_5_H_12_), macromolecule alkenes (e.g., C_6_H_12_, C_5_H_10_), and small-molecule alkynes (e.g., C_2_H_2_). The T _autoignition_ of C_5_H_12_ is lowest at 260°C. When there are no forced ignition sources, there are two typical situations:

a) When a cell erupts, the substances with T _auto-ignition_ lower than T _eruption_ are easy to autoignite first (e.g., macromolecular alkanes, macromolecular alkenes, and small molecular alkynes), and then, they ignite the substances with T _auto-ignition_ higher than T _eruption_ (e.g., CO, H_2_, small molecular alkanes, macromolecular alkynes, benzene, and electrolyte), as shown in Figure 8A.
b) If the CEGs are cooled below the minimal value of autoignitions of all components in the CEG (T _autoignition, min_) of ∼260°C, autoignition will not occur, as shown in Figure 8B.

Essentially, CEGs are mixtures of as many as 33 components. The gas mixtures can exhibit different characteristics (Bi et al., 2015) such as c_CEG, ignition_, c_O2, ignition_, and T _ignition_. However, no method has been found to accurately predict the T _ignition_ of the mixture. The T _ignition_ of the mixture is generally between the average T _ignition_ and lowest T _ignition_ of the components (Bi et al., 2015) and is strongly affected by the component with the lowest T _ignition_. Therefore, we used the lowest T _flash_ or T _autoignition_ of components of the CEG to characterize its T _ignition_. This is a method commonly used in combustion science and includes evaluation of the T _autoignition_ of the diesel-natural gas (NG) mixture in diesel-NG dual-fuel engines by the T _autoignition_ of diesel (Rosha et al., 2018).

The T _ignition_ of the mixture is also affected by the concentration of each component, particularly those with larger contents (Bi et al., 2015). Table 1 and Figure 7 show that the concentrations of CO, H_2_, and CH_4_ in CEGs are relatively large, with CH_4_ having the lowest T _flash_ among the 33 CEG components. Therefore, the analysis of forced ignition in this study is credible. Table 1 and Figure 8 show that the substances with the lowest T _autoignition_, such as C_5_H_12_, and C_5_H_10_, have low concentrations. However, according to the thermal ignition theory, even a relatively small amount of a substance can play a leading role in the ignition process. For example, the ignition of a premixed main charge containing gaseous fuel (more than 98% of the total fuel energy) occurs through direct injection of a small amount of diesel fuel (usually 0.5 to 2% of the total fuel energy) in a micro-pilot dual-fuel engine (Park et al., 2021). Diesel is a complex mixture of hydrocarbons containing 10–22 carbon atoms, and its T _autoignition_ is 254°C–285°C. Gases having a high T _autoignition_ include NG, which contains mainly CH_4_, C_2_H_6_, C_3_H_8_, C_4_H_10_, N_2_, and CO_2_; biogas, which contains mainly CO, CO_2_, CH_4_, and H_2_; H_2_; and others. This ignition process is strongly similar to that of the CET. Therefore, the analysis of autoignition in this study has certain reference value for evaluating the temperature boundary of the CEG. In particular, to leave a safe interval in the design target temperature to avoid fire, it is meaningful to use the lowest T _ignition_ among the CEG components to evaluate the T _ignition_ of the CEG.

**Figure 9:**
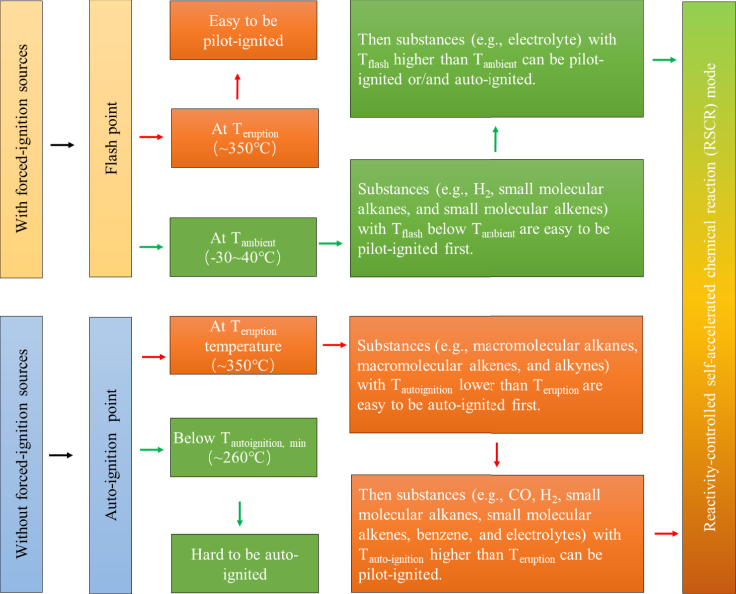
CEG ignition mode The ignition process of a cell belongs to the self-accelerating reaction mode, which is controlled by the reaction activity.

In short, when there is a forced ignition source, CEGs are prone to ignite regardless of the temperature, and the substances with a low T _flash_ (e.g., CO, hydrogen, small molecular alkanes, and small molecular olefins) play a leading role in the ignition process. When there are no forced ignition sources, CEGs are prone to autoignition at the T _eruption_, and the substances with a low T _autoignition_ (e.g., macromolecular alkanes, macromolecular alkenes, and small molecular alkynes.) play a leading role in the ignition process. If the CEG temperature is cooled below the T _autoignition, min_, autoignition will not occur. Therefore, the ignition process of a cell belongs to the self-accelerating reaction mode, which is controlled by the reaction activity, as shown in Figure 9. The CEG ignition mode can be controlled by changing the CEG temperature and ignition sources, i.e., reactivity-controlled self-accelerated chemical reaction mode (Li et al., 2019a).

### Significance of this research

The research results of this paper can provide guidance for cell selection, battery pack design, and safety design.

a) According to c_CEG, ignition_ and/or c_O2, ignition_, the following questions can be answered. Which cell type is safer? What is the right SOC value for cell storage? What is the CEG/O_2_ concentration value above which there is a possibility of fire? How much inert gases should be filled in a battery pack to ensure it does not ignite after eruption? How many cells experiencing thermal runaway can make the O_2_ concentration below c_O2, ignition_ by consuming the O_2_ inside a battery pack?
b) The research results related to T _ignition_ point out the importance of controlling the sources of forced ignition. They also show that when there are no ignition sources, the CEG temperature can be lowered to the T _auto-ignition_ (∼260°C) to avoid fires, providing a reference for thermal management design. In addition, the relevant results of this part also indicate the ignition mode of CEGs, laying a foundation for further research on related mechanisms.

The above results are only the most important ones. In short, through the analysis of the three fire boundaries, the occurrence of fire can be avoided when any one of the boundaries is avoided. According to the research results of this paper, a variety of solutions can be designed to avoid the occurrence of fire.

### Conclusions

In this study, the three fire boundaries, which are c_CEG, ignition_, c_O2, ignition_, and T _ignition_, were theoretically analyzed based on the CEG identification results of 29 thermal runaway tests in inert atmosphere. The main conclusions were summarized as follows:

(1) c_CEG, ignition_ decreases and then remains almost unchanged with the increase in SOC for the LFP_1.1 Ah (2015) and the NCA_3.35 Ah (2015) cells. For the LCO_2.1 Ah cell, with the increase in the SOC, c_CEG, ignition_ first increases and then decreases. The respective values of c_CEG, ignition_ for the LCO, LFP, NCA, and NMC cells are 4.4%–6.2%, 7.7%–36.6%, 5.4%–12.0%, and 3.9%–3.9%, respectively, which indicates that the order of c_CEG, ignition_ from high to low is LFP > NCA > LCO > NMC.
(2) c_O2, ignition_ does not significantly change for the LFP_1.1 Ah (2015) cell with the increase in the SOC. It decreases at the discharged stage but remains almost unchanged at the fully and overcharged stages for both NCA_3.35 Ah (2015) and LCO_2.1 Ah cells. The respective values of c_O2, ignition_ for the LCO, LFP, NCA, and NMC cells are 12.7%–14.9%, 8.9%–10.2%, 7.6%–11.0%, and 10.0%–15.1%, respectively, which indicates that the order of c_O2, ignition_ from high to low is LCO > NMC > LFP > NCA.
(3) When there is a forced ignition source, CEGs are prone to ignite regardless of the CEG temperature, and the substances with low T _flash_ play a leading role in the ignition process. When there are no forced ignition sources, CEGs are prone to autoignite at T _eruption_, and the substances with low T _autoignition_ play a leading role in the ignition process. When the CEG temperature is cooled below T _auto-ignition_ (∼260°C) of the CEG components, autoignition does not occur. The CEG ignition mode can be controlled by changing the CEG temperature and ignition sources.

### Limitations of the study

The release process of cell gas is a dynamic process, which is not considered in this study. In further research, the dynamic process of the cell fire boundary can be analyzed by computational fluid dynamics.

### Resource availability

#### Lead contact

Further information and requests should be directed to and will be fulfilled by the lead contact, Zhenhai Gao (gaozh@jlu.edu.cn).

#### Materials availability

This study did not generate any new materials.

### Data and code availability

Any data utilized in this study can be found in the main manuscript and supplemental information.

## Methods

All methods can be found in the accompanying transparent methods supplemental file.

## Supplemental information

Document S1. Transparent methods
