# Supplementary material for: Fire boundaries of lithium-ion cell eruption gases caused by thermal runaway
Source: iScience. 2021 Apr 7;24(5):102401. doi: 10.1016/j.isci.2021.102401 (PMC8102908; doi:10.1016/j.isci.2021.102401)
Supplement: Supplementary file 1 — Document S1. Transparent methods [file mmc1.pdf]

iScience, Volume *24*

Supplemental information

Fire boundaries of lithium-ion cell eruption gases caused by thermal runaway

Weifeng Li, Shun Rao, Yang Xiao, Zhenhai Gao, Yupeng Chen, Hewu Wang, and Minggao Ouyang

Transparent Methods

Calculation model of minimum CEG concentration required for ignition

The minimum CEG concentration required for ignition refers to the LFL of CEGs. When the CEG concentration is lower than a certain value, it is too lean to ignite. The calculation of $c_{CEG,ignition}$, i.e., LFL, is based on Le Chatelier's mixing rule (Chatelier, 1891; Mashuga et al, 2000), as shown in Equation 1.

$$c_{CEG,ignition} = LFL = \frac{1}{\sum_{i=1}^{n}\frac{x_i}{LFL_i}} \times 100\% \quad \text{(Equation 1)}$$

where LFL_i refers to the LFL of component i in CEG, and x_i refers to the volume percentage of component i in CEG.

During the calculation, the inert gas in CEG is considered to get a more accurate value of the flammability limit (Li, 1998; Tian et al., 2006; Wu et al., 1994)

Calculation model of minimum oxygen concentration required for ignition

The minimum O_2 concentration required for ignition refers to the oxygen concentration in the CEG-air mixture at the UFL (i.e., the CEG concentration in the mixture), as shown in Equation 2. When the oxygen concentration is lower than this value, the oxygen is too lean to support ignition. UFL has a similar calculation method to that of LFL, as shown in Equation 2.

$$c_{O_2,ignition} = (1 - UFL) * c_{O^2\ in\ air} = (1 - \frac{1}{\sum_{i=1}^{n}\frac{x_i}{UFL_i}} \times 100\%) * c_{O^2\ in\ air} \quad \text{(Equation 2)}$$

where $c_{O^2\ in\ air}$ refers to the O_2 volume percentage in air, UFL_i refers to the UFL of component i in CEG, and x_i refers to the volume percentage of component i in CEG.

Supplemental References

Chatelier, L. (1891). Estimation of Firedamp by Flammability Limits (Annals of mines).

Li, D. (1988). Calculation of explosive concentration limit of flammable gas. Chem. Des. Commun. 14, 63-65.

Mashuga, C.V., and Crowl, D.A. (2000). Derivation of Le Chatelier's mixing rule for flammable limits. Process Saf. Prog. 19, 112–117.

Tian, G., Yu, C., and Li, X. Study on calculation on method of gas explosion limits, Gas Heat 26 (2006) 29-33.

Wu, J., Kong, Q., and Wang, B. (1994). Theoretical calculation method for the explosion limit of mixed gas. Oil Gas Storage Trans.13, 10-12.
